# Determinants of work-related musculoskeletal disorders among coal miners in Jining, China: development of a predictive risk model

**DOI:** 10.3389/fpubh.2026.1729879

**Published:** 2026-02-05

**Authors:** Jiali Li, Xuemei Zhang, Yuchen Li, Wenwen Ding, Wenhua Duan, David Lim, Yumin Liang, Zhihui Feng

**Affiliations:** 1 Department of Labor Hygiene and Environmental Hygiene, School of Public Health, Cheeloo College of Medicine, Shandong University, Jinan, China; 2 Jining City Centre for Disease Control and Prevention, Jining, China; 3 Faculty of Health, University of Technology Sydney, Ultimo, NSW, Australia

**Keywords:** coal miners, machine learning, predictive model, risk factors, work-related musculoskeletal disorders

## Abstract

**Background:**

Work-related musculoskeletal disorders (WMSD) are highly prevalent among coal miners and pose a significant threat to occupational health. Understanding the underlying risk factors and developing a predictive model for WMSD risk can help to mitigate WMSD.

**Objective:**

To identify key determinants of WMSD among coal miners in Jinang, China, and construct a predictive model to assess risk.

**Methods:**

One thousand four hundred nine coal miners from two coal mining companies were surveyed using the modified Chinese Muscle Questionnaire (CMQ). Prevalence rates and risk factors were assessed using logistic regression. Machine learning algorithms were applied to construct the predictive model.

**Results:**

The 12-month overall prevalence of WMSD was 82%, with the neck (59.5%), shoulders (53.4%), and lower back (46.5%) being the most affected. Eight variables, including smoking behaviors, perceived health status, and uncomfortable working posture, were significantly associated with WMSD (*p* < 0.05). The neural network model achieved the highest performance (area under the curve: 0.886 on training and 0.704 on test). The fused model outperformed individual models in the final stacking integration learning.

**Conclusion:**

Work-related musculoskeletal disorders are highly prevalent among Chinese coal miners and are influenced by personal and work-related factors. Machine learning models, particularly ensemble approaches, offer promise for risk prediction and targeted prevention.

## 1 Introduction

Work-related musculoskeletal disorders (WMSD) are a category of cumulative injuries affecting the musculoskeletal system and refer to injuries and disorders that occur to muscles, nerves, tendons, ligaments, joints, cartilage, and intervertebral disks (1). WMSD symptoms usually develop gradually over a period of time rather than being caused by a single traumatic event (2). WMSD can be caused by prolonged maintenance of specific static positions or postures, and by repetitive movements required by specific job tasks. It mainly affects the neck, shoulders, limbs, and back and lumbar region (3). WMSD not only affects the quality of life of individual workers but also imposes a significant economic burden on society through reduced operational efficiency (reduced productivity, increased absenteeism), affected workforce stability (increased turnover, early retirement), increased costs (medical expenses, insurance and compensation), and psychological distress. WMSD is one of the world’s most pressing health challenges (4). The recent Global Burden of Disease Study 2019 estimated that approximately 1.71 billion people worldwide are affected by musculoskeletal disorders, this represents the most significant contributor to overall disease prevalence globally (5). WMSDs rank second among occupational diseases in many European and North American countries. In the United States (US), WMSDs represent the fastest-growing category of occupational illness, resulting in annual workers’ compensation and insurance costs amounting to tens of billions of US dollars. In Europe, approximately 30% of workers report experiencing back pain, underscoring the substantial burden of musculoskeletal conditions in the workforce. In Japan, WMSDs constitute the largest category of work-related diseases, accounting for one-third or more of all registered occupational diseases (6). Evidence from China similarly indicates a high prevalence of WMSDs in occupational groups. In manufacturing settings, a meta-analysis focusing on China’s automobile manufacturing industry estimated a pooled 12-month WMSDs prevalence of 53.1% (7).

While China has pledged to achieve carbon neutrality by 2060, coal remains central to the country’s energy infrastructure. As the world’s largest producer of raw coal, China employs millions of workers across coal mining, processing, washing, and beneficiation sectors (8). Coal miners represent a distinct occupational group due to their exposure to high-stress conditions and harsh environments. Their work involves prolonged exposure to dust, chemical agents, physical strain, and biological hazards, factors that collectively contribute to psychological stress, job fatigue, and burnout (9). Among the health challenges faced by this workforce, WMSD has emerged as a particularly pressing concern (10). Globally, coal miners exhibit a notably high prevalence of WMSD, underscoring the need for targeted occupational health interventions (11).

While several factors are known to be associated with WMSD, their relative impact can vary depending on region, occupational, and individual differences. These risk factors have different effects on the body, leading to diverse manifestations of WMSD (12). Research on predictive models of WMSD pertaining to coal mining is limited (13). Advancement in technology, such as machine learning, offers an opportunity to harness data to better predict the determinants of WMSD.

In this study, a modified version of the musculoskeletal questionnaire was used to identify the risk factors for WMSDs. Machine learning algorithms were used to construct a prediction model for WMSD based on the collected data. The goal is to provide a scientifically grounded approach for the prevention and protection of workers’ health.

## 2 Materials and methods

### 2.1 Study design and setting

This study employed a cross-sectional design to investigate the prevalence and determinants of work-related musculoskeletal disorders (WMSDs) among frontline coal mining workers and to develop predictive models for WMSD risk using both traditional statistical methods and machine learning algorithms. This study was reported in accordance with the Strengthening the Reporting of Observational Studies in Epidemiology (STROBE) statement: guidelines for reporting observational studies (14). The cross-sectional survey was conducted between January and February 2024 in two coal mining companies in Jining City, Shandong Province, China.

### 2.2 Participants

#### 2.2.1 Comparability of participants

The study population comprised frontline operational workers employed at two coal mining enterprises in Jining City, Shandong Province. The two participating coal mining companies were broadly comparable in terms of organizational structure, each comprising similar production departments, frontline job categories and descriptions, and occupational health and safety management systems. With respect to job responsibilities and work equipment, workers in both companies were primarily engaged in coal extraction, tunneling, and related underground auxiliary operations. The nature of the work and the production equipment used were consistent with standard practices in the coal mining industry and were therefore highly comparable. Regarding working hours, both companies operated a shift-based system; however, the schedules differ: a 40-h work week in one company and a 36-h work week in the other. Overall, despite these differences in weekly working hours, the two companies were comparable in terms of job characteristics and descriptions, working environments, and potential occupational exposure profiles.

#### 2.2.2 Eligibility criteria and participant selection

Frontline operational workers were eligible for inclusion if they met the following criteria:

- in full-time employment for more than 1 year.
- ≥18 years old.
- No language or communication barriers.
- Provided express and informed consent to participate in this study.

Workers with musculoskeletal disorders due to infectious diseases, rheumatism, malignant tumors, congenital disorders and other non-occupational causes were excluded.

Professional staff from Shandong University School of Public Health and Jining Center for Disease Control and Prevention were responsible for subject selection. These investigators possessed backgrounds in public health and preventive medicine, along with extensive experience in questionnaire surveys. Prior to the survey commencement, investigators received systematic training on the study objectives, inclusion criteria, and data collection procedures. All eligible active-duty personnel were consecutively invited to participate. Prior to formal questionnaire distribution, all workers received a unified briefing explaining the study objectives, detailing the questionnaire completion process, and outlining relevant precautions. Participants voluntarily enrolled after receiving full informed consent and independently completed the questionnaires. To ensure data quality and procedural standardization, the questionnaire incorporated automatic logic checks and mandatory fields, with real-time monitoring of completion progress. During completion, on-site investigators addressed any participant inquiries without influencing or interfering with responses, thereby minimizing potential selection and information biases.

#### 2.2.3 Ethical considerations

Ethical approval was obtained independently from the School of Public Health, Shandong University (LL20231201), in accordance with the Declaration of Helsinki on the responsible and ethical conduct of human research.

### 2.3 Sample size

The minimum sample size required was 483 (based on *α* = 0.05 and 𝛿 = 0.03, with a further 20% non-response rate). The final sample size obtained was 1,409. This larger sample size was expected to improve the robustness and stability of the multivariable models.

### 2.4 Instrument and data collection

#### 2.4.1 Questionnaire

Data were collected using a validated and established, modified Chinese Musculoskeletal Questionnaire (CMQ), adapted from the Nordic Musculoskeletal Questionnaire (15, 16). The CMQ has been previously tested for validity and reliability in various Chinese occupational groups (3, 4).

The questionnaire consisted of three sections:

- Demographic factors (gender, age, years of work experience, and marital status),
- Self-reported musculoskeletal symptoms (body parts, frequency, intensity and duration),
- Occupational exposure-related factors.

#### 2.4.2 Quality control

Before data collection, trained investigators provided a standardized briefing to participants, explaining the study objectives, questionnaire content, and completion procedures. The survey was conducted using a face-to-face electronic questionnaire developed on the Wenjuanxing platform, with participants completing the questionnaire independently by scanning a QR code on their mobile devices. Communication between participants was not permitted during completion, and investigators offered standardized guidance without interfering with responses. To ensure data quality, the questionnaire incorporated mandatory fields and automated logical consistency checks, and completion progress was monitored in real time. Any questions raised during the survey were addressed by trained investigators to facilitate accurate and complete data collection while minimizing information bias.

### 2.5 Data analysis

Descriptive statistics were used to summarize the basic characteristics. Continuous variables were reported as medians and interquartile ranges, while categorical variables were expressed as frequencies and percentages. Differences between groups were compared using Pearson’s chi-square (*χ*^2^) test or Fisher’s exact probability method, as appropriate. Multivariate logistic regression analysis was employed to identify independent risk factors for WMSD. Variables with statistically significant associations in univariate analyses (*p* < 0.05) were included in the regression model.

### 2.6 Machine learning algorithm

Drawing on prior literature and the application of machine learning methods to occupational health risk prediction, this study employed multiple supervised learning algorithms to develop predictive models for WMSD risk among coal miners. All models were constructed after completing data preprocessing procedures, including variable encoding, standardization, and handling of missing values. Model development and analysis were conducted using the R software environment (version 4.1.2). To ensure reproducibility, a unified random seed (seed = 123) was applied across all model training processes.

A total of six machine learning algorithms were included in this study: random forest (RF), decision tree (DT), neural network (NN), Naïve Bayes (NB), support vector machine (SVM), and k-nearest neighbors (K-NN). WMSD occurrence was specified as the dependent variable, and feature variables identified by logistic regression were used as independent variables. Model outputs were expressed as individual predicted probabilities of WMSD occurrence (ranging from 0 to 1). Risk stratification was performed based on these predicted probabilities, with consistent thresholds applied across all models to define high risk (>0.65), low risk (<0.35), and intermediate risk categories.

#### 2.6.1 Model introduction and parameter settings

##### 2.6.1.1 Random forest model (RF)

A random forest model is a supervised learning algorithm based on ensemble learning, which reduces the risk of overfitting and improves model generalizability by constructing multiple decision trees and aggregating their prediction results. In this study, a classification model was developed using the randomForest package in R. The number of trees was set to 500 (ntree = 500), and the number of variables randomly selected at each node split was set to 8 (mtry = 8). This approach is well-suited for high-dimensional data, demonstrates strong robustness to noise and missing values, and provides measures of variable importance to facilitate the identification of key influencing factors.

##### 2.6.1.2 Decision tree model (DT)

The decision tree model constructs a tree-like structure by recursively partitioning the feature space, resulting in an intuitive and easily interpretable model. In this study, a decision tree model was developed using the rpart package in R. The complexity parameter was set to 0.01 (cp = 0.01) to control tree growth, the maximum tree depth was limited to 5 (maxdepth = 5), and the minimum number of samples required for a node split was set to 20 (minsplit = 20). In addition, 10-fold cross-validation was applied in the rpart framework to enable automatic cost-complexity pruning, and the subtree with the minimum cross-validation error was selected to mitigate overfitting.

##### 2.6.1.3 Neural network model (NN)

The neural network model was used to capture nonlinear relationships among variables. In this study, a single-hidden-layer neural network was constructed using the nnet package within the caret framework in R. The number of neurons in the hidden layer was set to 8 (size = 8), and L2 regularization was applied with a decay parameter of 0.1 (decay = 0.1) to mitigate overfitting. The maximum number of training iterations was set to 200 (maxit = 200). During model training, parallel computing was enabled to improve computational efficiency, and model convergence was monitored throughout the training process.

##### 2.6.1.4 Naïve Bayes model (NB)

The Naïve Bayes model performs probabilistic inference based on Bayes’ theorem and is well suited for high-dimensional and small-sample datasets. In this study, the NB model was constructed using the e1071 package in R, with Laplace smoothing applied (laplace = 1) to address zero-probability issues. For continuous variables, kernel density estimation was used by default to preserve the underlying distributional characteristics.

##### 2.6.1.5 Support vector machine model (SVM)

The support vector machine (SVM) model performs classification by constructing an optimal separating hyperplane and is particularly effective for handling nonlinear relationships. In this study, the SVM model was developed using the e1071 package in R with a radial basis function kernel (kernel = “radial”). The regularization parameter was set to 1 (cost = 1), and the kernel parameter was set to 1 (gamma = 1). Probability estimation was enabled (probability = TRUE) to generate individual predicted probabilities of WMSD occurrence.

##### 2.6.1.6 K-nearest neighbors model (K-NN)

The k-nearest neighbors (K-NN) algorithm is an instance-based, nonparametric supervised learning method. In this study, a K-NN classification model was constructed using the caret package in R. Continuous variables were centered and scaled prior to model training to eliminate the influence of scale differences, and Euclidean distance was used as the similarity metric. The optimal number of neighbors was selected through automated tuning (tuneLength = 10). Model performance and generalizability were evaluated using 10-fold cross-validation.

##### 2.6.1.7 Stacking ensemble model

Ensemble learning was applied to improve the predictive accuracy and generalizability of the WMSD risk model. A stacking ensemble strategy was implemented, in which six heterogeneous machine learning algorithms (neural network, random forest, decision tree, Naïve Bayes, support vector machine, and k-nearest neighbors) were used as first-level base learners and trained using the caret package in R. Model training employed three-fold cross-validation combined with five bootstrap resampling iterations to reduce overfitting, with the area under the receiver operating characteristic curve (AUC) used as the primary performance metric. Predicted probabilities from the base models were used as meta-features and integrated using a generalized linear model at the second level.

#### 2.6.2 Validation and evaluation of predictive models

All models were trained and evaluated using the same training and test datasets. The dataset was randomly split into a training set (70%) and a test set (30%) using a fixed random seed (seed = 123). Model training and parameter optimization were performed exclusively on the training set, while the test set was used solely for validation and performance evaluation. This approach was adopted to minimize overfitting and to ensure an unbiased assessment of model performance.

The predictive performance of the machine learning models was evaluated on the test dataset. The primary performance metric was the area under the receiver operating characteristic curve (AUC), and additional metrics, including accuracy, precision, recall, and F1-score, were also calculated. Accuracy, precision, recall, and F1-score were derived from the binary confusion matrix. Detailed definitions of the confusion matrix and calculation formulas for each metric are provided in the Supplementary Table S4 and Supplementary Equations 1–4.

##### 2.6.2.1 Accuracy

Accuracy refers to the proportion of correctly predicted samples out of the total number of samples. It serves as a fundamental metric for evaluating a model’s overall performance and is suitable for datasets with balanced class distributions. However, when data exhibits class imbalance, accuracy may fail to fully reflect the model’s actual performance.

##### 2.6.2.2 Precision

Accuracy refers to the proportion of samples predicted as positive cases by the model that are actually positive cases. Accuracy reflects the reliability of the model in predicting positive cases. In WMSD risk prediction, high accuracy means the model can reduce false positives, thereby lowering unnecessary intervention costs.

##### 2.6.2.3 Recall

Recall refers to the proportion of samples that are actually positive cases and are correctly predicted as positive by the model. Recall reflects the model’s ability to identify positive case samples. In WMSD risk prediction, a high recall indicates that the model can identify as many high-risk workers as possible, avoiding underreporting and thereby reducing the risk of occupational disease occurrence.

##### 2.6.2.4 F1-score

F1 score is the harmonic mean of precision and recall, providing a balanced measure of both accuracy and sensitivity. It is particularly useful for evaluating model performance when positive and negative samples are imbalanced, offering a more comprehensive assessment. The F1 score is a synthetic metric that reflects the overall effectiveness of a model more accurately than precision or recall alone, especially when dealing with imbalanced data. It ranges from 0 to 1, with higher values indicating better performance in both precision and recall.

##### 2.6.2.5 Receiver operating characteristic curve and AUC

The ROC curve is a graph plotting the false positive rate (FPR) on the *x*-axis and the true positive rate (TPR) on the *y*-axis, illustrating the relationship between true positive and false positive rates. AUC refers to the Area Under the Receiver Operating Characteristic curve, i.e., the area beneath the ROC curve, with values ranging from 0 to 1. An AUC value of 0.5 indicates that the model’s predictive capability is equivalent to random guessing, signifying poor predictive ability and no practical application value. An AUC value between 0.5 and 0.7 indicates average predictive performance; an AUC value > 0.7 signifies good predictive performance with high practical value. AUC reflects the model’s overall performance across different classification thresholds. The closer the AUC value is to 1, the stronger the model’s classification capability.

## 3 Results

### 3.1 Participant demographics

Of the 1,409 participants, 1,308 (92.8%) were male. The predominant age group was 30–40 years old (*n* = 965, 58%). Most participants (*n* = 783, 55.6%) had a normal BMI (18.5 to 25 kg/m^2^), while 539 (38.2%) were classified as overweight (25 kg/m^2^ ≤ BMI < 30 kg/m^2^) and 67 (4.8%) were classified as obesity (BMI ≥ 30 kg/m^2^). In terms of educational attainment, 808 (48.6%) of the participants had completed high school, 440 (26.4%) had completed junior college, and only 294 (17.7%) held a bachelor’s degree. The majority had 10–20 years of work experience (*n* = 877, 62.2%), followed by those with >20 years of service (*n* = 314, 22.4%). Nearly half of the participants reported occasional physical activity (*n* = 668, 47.4%), while 615 (43.6%) reported no regular exercise. Additional demographic characteristics are presented in Table 1.

**Table 1:** Demographic characteristics of the participants.

Variables categories		*N*	Percentage (%)
Gender	Male	1,308	92.8
	Female	101	7.2
Age (years)	20 ~ 30	93	6.6
	30 ~ 40	812	57.6
	40 ~ 50	425	30.2
	>50	79	5.6
Body Mass Index (kg/m^2^)	<18.5	20	1.4
	18.5 ~ 25	783	55.6
	25 ~ 30	539	38.2
	≥30	67	4.8
Hand	Right	1,266	89.9
	Left	143	10.1
Education	Junior high school and below	104	7.4
	High school	736	52.2
	Junior college	363	25.8
	Bachelor degree	202	14.3
	Master degree or above	4	0.3
Marital status	Unmarried	66	4.7
	Married and living with spouse	1,311	93
	Married but separated	32	2.3
Monthly income (RMB*)	≤3,000	50	3.5
	3,001–5,000	422	30
	5,001–10,000	773	54.9
	>10,000	164	11.6
Physical exercise	No	615	43.6
	Occasionally	668	47.4
	2 ~ 3 times/month	27	1.9
	1 ~ 2 times/week	50	3.5
	>2 times/week	49	3.5
Smoking behavior	Non-smoker	571	40.5
	Occasional smoking	361	25.6
	Regular smoking	433	30.7
	Quit smoking	44	3.1
Length of service in this job category	<5	415	29.5
	5 ~ 10	318	22.6
	10 ~ 15	351	24.9
	>15	325	23.1
Length of employment	≤10	218	15.5
	10 ~ 20	877	62.2
	>20	314	22.3
Total		1,409	100

*RMB in table refers to Renminbi, the official legal currency of China.

### 3.2 Prevalence of WMSD

In total, 1,156 (82%) participants reported experiencing work-related musculoskeletal pain or discomfort in the past 12 months. Multisite (2 or more) WMSD were more prevalent (86.3%) than single-site (13.7%). The most commonly affected areas were the neck (59.5%), shoulder (53.4%) and low back (46.5%) (Table 2).

**Table 2:** Prevalence of WMSD by body region.

Body parts	Positive case (n)	Prevalence (%)
Neck	839	59.5
Shoulder	752	53.4
Upper back	377	26.8
Low back	655	46.5
Elbow	571	40.5
Wrist/hand	620	44
Hip/Thigh	484	34.4
Knee	598	42.4
Ankle/foot	485	34.4

### 3.3 Occupational risk factor

#### 3.3.1 Univariate analysis

Univariate analyses were performed using Pearson’s *χ*^2^ test and the *χ*^2^ test for trend on 9 demographic variables and 49 work-related variables to identify variables associated with work-related musculoskeletal disorders (WMSDs). The results for demographic characteristics are summarized in Table 3, and the complete univariate analysis results are presented in Supplementary Table S1. The *χ*^2^ test results indicated that, among individual-level factors, employment length was significantly associated with WMSD prevalence, with the risk increasing as years of employment increased. Married workers exhibited a higher prevalence of WMSDs than unmarried workers. In addition, workers who regularly engaged in physical activity had a lower prevalence of WMSDs compared with those who did not or only rarely participated in physical activity. Regular smokers showed a higher prevalence of WMSDs than non-smokers, occasional smokers, and former smokers. In contrast, sex, age, body mass index (BMI), educational level, and monthly income were not significantly associated with WMSD occurrence.

**Table 3:** Comparison of WMSD with different demographic characteristics.

Factors	*N*	Positive case (n)	Prevalence (%)	*χ* ^2^	*P*
Length of service in this job category					
<5	415	325	78.3	10.793	0.013
5 ~ 10	318	253	79.6		
10 ~ 15	351	299	85.2		
>15	325	279	85.8		
Length of employment					
≤10	218	152	69.7	28.592	<0.001
10 ~ 20	877	731	83.4		
>20	314	273	86.9		
Marital status					
Unmarried	66	43	62.5	13.471	0.001
Married and living with spouse	1,311	1,087	82.9		
Married but separated	32	26	81.25		
Physical exercise					
No	615	534	86.8	20.812	<0.001
Occasionally	668	527	78.9		
2 ~ 3times/month	27	23	85.2		
1 ~ 2times/week	50	38	76		
> 2 times/week	49	34	69.4		
Smoking behavior					
Non-smoker	571	455	79.7	14.111	0.003
Occasional	361	285	78.9		
Regular	433	380	87.8		
Quit smoking	44	36	81.8		

In addition, the occurrence of WMSDs was significantly associated with multiple work-related factors, including perceived health status, prolonged standing, prolonged kneeling, prolonged trunk bending, prolonged neck flexion, prolonged knee bending, high-frequency repetitive operations per minute, frequent repetition of the same back movements, repetitive wrist flexion/extension, uncomfortable working postures, repetitive operations performed multiple times per minute, carrying heavy loads (more than 20 kg per lift), operating with hands or arms, overtime work, feeling cold at work, exposure to wind or temperature changes in the working environment, and insufficient rest time. For example, workers who frequently stood for long periods during work had a markedly higher prevalence of WMSDs than those in other work categories, whereas workers who rarely or never stood for prolonged periods had a lower prevalence of WMSDs. To improve clarity and conciseness, Table 4 presents only variables with *p* values less than 0.001 in the univariate analyses. The complete results of the *χ*^2^ tests for all work-related variables are provided in Supplementary Table S2.

**Table 4:** Comparison of WMSD with different work-related factors.

Factors	*N*	Positive case	Prevalence (%)	*χ* ^2^	*P*
Perceived health status					
Perfect	462	308	66.7	111.438	<0.001
Fine	832	741	89.2		
Poor	103	96	93.2		
Very poor	12	11	91.7		
Keep standing for long hours					
Never	236	181	76.7	38.375	<0.001
Occasionally	253	185	73.1		
Frequently	362	292	80.7		
Very Frequently	558	498	89.2		
No	743	577	77.7	20.525	<0.001
Yes	666	579	86.9		
Keep bowing the head for long hours					
No	786	603	76.7	34.235	<0.001
Yes	623	553	88.8		
Keep bending knees for long hours					
No	751	582	77.5	22.573	<0.001
Yes	658	574	87.2		
Repeat operation many times a working minute					
No	594	438	73.7	48.099	<0.001
Yes	815	718	88.1		
Frequent repetition of the same movement on the back					
No	459	340	74.1	29.353	<0.001
Yes	950	816	85.9		
Bending wrist up/down					
No	403	292	72.5	35.218	<0.001
Yes	1,006	864	85.9		
Work in uncomfortable postures					
Never	359	251	69.9	65.717	<0.001
Occasionally	528	429	81.3		
Frequently	333	303	91		
Very Frequently	189	173	91.5		
Operate repetitively for multiple times per minute					
Never	281	203	72.2	56.769	<0.001
Occasionally	429	327	76.2		
Frequently	395	347	87.8		
Very Frequently	304	279	91.8		
Carry heavy objects (more than 20 kg each time)					
Never	355	262	73.8	34.335	<0.001
Occasionally	433	346	79.9		
Frequently	331	289	87.3		
Very Frequently	290	259	89.3		
Operate with hands or arms					
Never	224	166	74.1	46.305	<0.001
Occasionally	289	210	72.7		
Frequently	419	353	84.2		
Very Frequently	477	427	89.5		
Overtime work					
No	725	549	75.7	40.49	<0.001
Yes	684	607	88.7		
Feeling cold, wind or temperature change at work					
No	283	192	67.8	48.467	<0.001
Yes	1,126	964	85.6		
Enough rest time					
No	939	832	88.6	82.252	<0.001
Yes	470	324	68.9		
Back position					
Upright	492	372	75.6	22.52	<0.001
Slightly curved	591	499	75.6		
Substantial bending	326	285	87.4		
Neck position					
Upright	460	339	73.7	35.701	<0.001
Slightly forward leaning	601	507	84.4		
Large forward lean	272	243	89.3		
Head tilted back	76	67	88.2		
Keep the neck in the same position for long hours					
No	613	475	77.5	15.29	<0.001
Yes	796	681	85.6		
Keep turning the head for long hours					
No	889	702	79	15.5	<0.001
Yes	520	454	87.3		
Keep wrist flexion for long hours					
No	623	471	75.6	31.461	<0.001
Yes	786	685	87.2		
No	495	377	76.2	17.923	<0.001
Yes	914	779	85.2		
Lower extremities do the same movement frequently					
No	610	465	76.2	24.687	<0.001
Yes	799	691	86.5		
Turn around often					
No	358	270	75.4	14.299	<0.001
Yes	1,051	886	84.3		
Carry heavy objects (more than 5 kg each time)					
Never	297	228	76.8	31.076	<0.001
Occasionally	376	288	76.6		
Frequently	356	297	83.4		
Very frequently	380	343	90.3		
Holding onto something in hand					
No	266	189	71.1	26.89	<0.001
Yes	1,143	967	84.6		
Shortage of staff in the sector					
No	429	302	70.4	56.803	<0.001
Yes	980	854	87.1		
Work for someone else					
No	972	774	79.6	12.401	<0.001
Yes	437	382	87.4		
Use vibrator at work					
Never	456	350	76.8	26.438	<0.001
Occasionally	425	338	79.5		
Frequently	279	250	89.6		
Very Frequently	249	218	87.6		
Wrist on the edge of hard, angular objects					
No	728	567	77.9	17.689	<0.001
Yes	681	589	86.5		

#### 3.3.2 Multivariate logistic regression screening for influential factors

Eight variables were identified as independent predictors of WMSDs. Seven determinants were associated with WSMD:

- Smoking (OR = 1.583; 95% CI: 1.039–2.412)
- Frequent multiple repetitive operations (OR = 1.672; 95% CI: 1.161–2.410)
- Prolonged head-down posture (OR = 1.639; 95% CI: 1.039–2.584)
- Uncomfortable working postures (OR = 1.912; 95% CI: 1.184–3.088)
- Prolonged knee bending (OR = 1.531; 95% CI: 1.002–2.339)
- Employed >20 years (OR = 2.364; 95% CI: 1.300–4.300)
- Self-perceived poor health status (OR = 4.034; 95% CI: 1.680–9.688)

Having sufficient rest time appeared to be a protective factor against WMSD (OR = 0.518; 95% CI: 0.355–0.757). Results are summarized in Table 5.

**Table 5:** Results of multivariate logistic regression describing the relationship between personal and work-related factors with WMSD.

Factors		ORs	95%CI		*P*
			Lower	Upper	
Smoking behavior	No				
	Regular	1.583	1.039	2.412	0.033
Perceived health status	Perfect				
	Fine	2.895	2.048	4.093	<0.001
	Poor	4.034	1.68	9.688	0.002
Work in uncomfortable postures	Never				
	Occasionally	1.912	1.184	3.088	0.008
Repeat operation many times a working minute	No				
	Yes	1.672	1.161	2.41	0.006
Enough rest time	No				
	Yes	0.518	0.355	0.757	0.001
Keep bowing the head for long hours	No				
	Yes	1.639	1.039	2.584	0.034
Keep bending knees for long hours	No				
	Yes	1.531	1.002	2.339	0.049
Length of employment (years)	<10				
	>20	2.364	1.3	4.3	0.005

Although sex and age did not reach statistical significance in the univariate analyses, both variables may act as potential confounders influencing the associations between multiple predictors and WMSD prevalence. To assess the potential confounding effects of age and sex, we conducted a sensitivity analysis by adding these two variables to the multivariable logistic regression model. The inclusion of age and sex did not materially alter the direction or magnitude of the main associations. The full results of this analysis are presented in Supplementary Table S3.

### 3.4 Construction of a risk prediction model for WMSD

#### 3.4.1 Construction and validation of a single model

**Figure 1:**
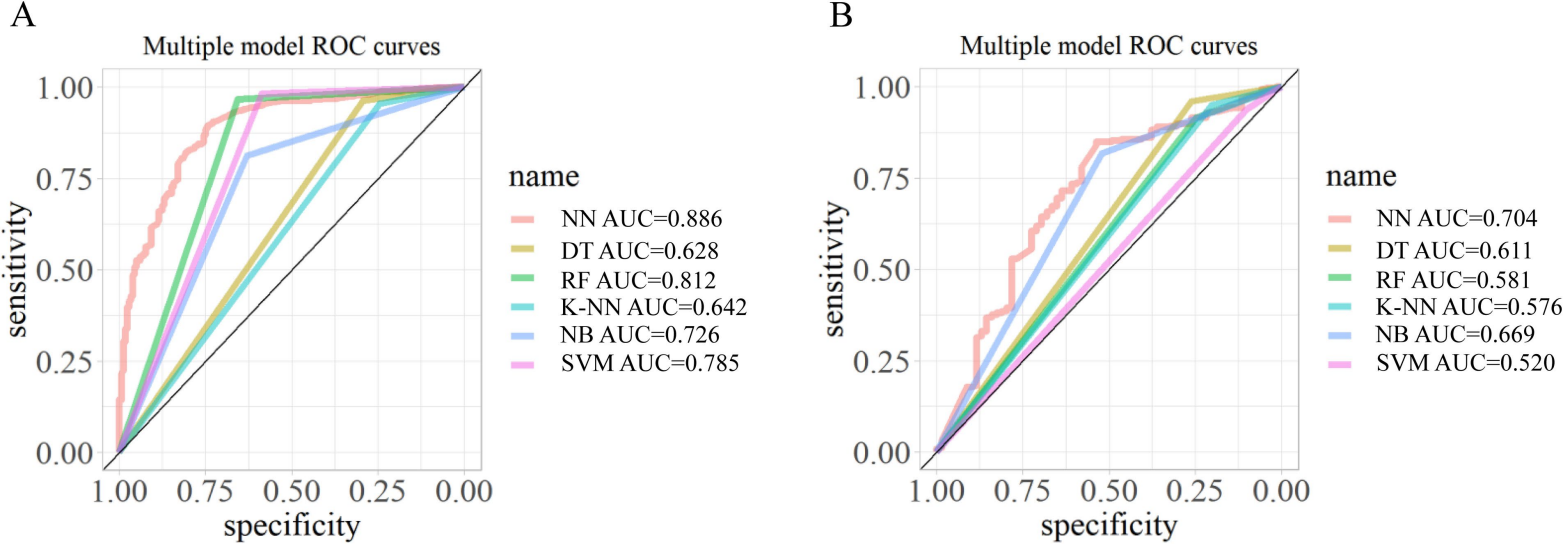
ROC curves for six base models: **(A)** ROC curve for training set and **(B)** ROC curve for test set.

The eight identified determinants included in the six machine learning models are: length of employment (years), smoking behavior, perceived health status, working in uncomfortable postures, repetitive operation, rest time, duration of a head bow, and knee bend. The comparison of the individual model’s accuracy, precision, recall, F1 score and AUC are presented in Tables 6–10 and Figure 1. In summary, the NN model has the highest AUC (0.886 training, 0.704 test), strong precision and F1 score. The NB model has stable performance across metrics. The RF model has high accuracy but lower recall in the test set. The SVM model reported high precision but inconsistent recall. The DT and K-NN models had moderate performance.

**Table 6:** Comparison of the accuracy of the training and test sets of the six models.

Algorithm	Training set		Test set	
	Mean	95%CI	Mean	95%CI
Random forest	0.909	(0.889, 0.926)	0.809	(0.755, 0.834)
Decision tree	0.838	(0.813, 0.860)	0.846	(0.808, 0.879)
Naïve Bayes	0.779	(0.752, 0.805)	0.768	(0.725, 0.808)
Neural network	0.865	(0.842, 0.886)	0.839	(0.801, 0.873)
Support vector machines	0.909	(0.889, 0.926)	0.801	(0.260, 0.838)
K-nearest neighbors	0.837	(0.812, 0.859)	0.837	(0.783, 0.858)

**Table 7:** Comparison of the precision of the training and test sets of the six models.

Algorithm	Training set	Test set
Random forest	0.966	0.918
Decision tree	0.963	0.960
Naïve Bayes	0.811	0.816
Neural network	0.966	0.949
Support vector machines	0.983	0.938
K-nearest neighbors	0.953	0.949

**Table 8:** Comparison of the recall value of the training and test sets of the six models.

Algorithm	Training set	Test set
Random forest	0.658	0.246
Decision tree	0.293	0.261
Naïve Bayes	0.641	0.522
Neural network	0.424	0.275
Support vector machines	0.587	0.101
K-nearest neighbors	0.332	0.261

**Table 9:** Comparison of the F1-score of the training and test sets of the six models.

Algorithm	Training set	Test set
Random forest	0.783	0.389
Decision tree	0.450	0.410
Naïve Bayes	0.716	0.637
Neural network	0.589	0.427
Support vector machines	0.735	0.183
K-nearest neighbors	0.492	0.409

**Table 10:** Comparison of the AUC of the training and test sets of the six models.

Algorithm	Training set	Test set
Random forest	0.812	0.581
Decision tree	0.628	0.611
Naïve Bayes	0.726	0.669
Neural network	0.886	0.704
Support vector machines	0.785	0.520
K-nearest neighbors	0.642	0.576

#### 3.4.2 Ensemble model

To enhance the overall predictive accuracy of the individual models, a stacking ensemble model was constructed using the six individual models and a generalized linear model as the meta-learner. The ensemble model achieved the highest AUC (0.759), outperforming all individual models. Results are shown in Table 11.

**Table 11:** Comparison of AUC values for each model after integration.

Algorithm	AUC
Ensemble	0.759
Neural network	0.746
Decision tree	0.65
Random forest	0.727
K-nearest neighbors	0.725
Support vector machine	0.708
Naïve Bayes	0.731

## 4 Discussion

Musculoskeletal disorders affect about one-fifth of the world’s population and are a leading cause of disability and reduced quality of life worldwide (5). WMSD is an emerging focus of public health and occupational health research due to its substantial impact on worker well-being and organizational productivity. These conditions are particularly prevalent in physically demanding industries, where repetitive motion, sustained awkward postures, and biomechanical strain are common. Beyond the individual burden, WMSD contributes to increased absenteeism, reduced work efficiency, and escalating healthcare and social insurance costs. As a result, identifying and predicting determinants of WMSD would aid in targeted interventions to mitigate risk and improve workforce sustainability.

In this study, the 12-month prevalence of WMSD among Chinese coal miners was 82%, with the neck, shoulders, and lower back being the most frequently affected sites. This prevalence was higher than that reported among workers in some manufacturing and service sectors (17, 18), but comparable to estimates from studies conducted in similar mining occupations (19). Previous epidemiological studies have consistently shown a higher prevalence of WMSDs among mining workers compared with the general industrial workforce (20). This pattern is likely related to sustained heavy physical workload, repetitive work tasks, and constrained working postures. Overall, differences in WMSD prevalence across regions appear to largely reflect variations in occupational workload and working conditions. The high prevalence of multisite WMSD (86.3%) further emphasizes the cumulative nature of these disorders in physically intensive occupations such as coal mining.

The etiopathology of WMSD involves a complex interplay of behavioral, occupational and environmental factors. Our findings indicate that the length of employment is a significant predictor of WMSD, consistent with prior research that associates prolonged occupational exposure with musculoskeletal injuries (7, 21, 22). This may be due to the compounding effect of aging with sustained physical exertion, which contributes to progressive tissue damage, inadvertently increasing the risk of injury and leading to WMSD. Our observations reinforce the understanding of WMSD as cumulative traumas disorders, often resulting from repetitive strain and biomechanical overload.

Regarding individual behavioral factors, our study found physical activity to be a protective factor against WMSD. Workers who engaged in regular exercise had a lower prevalence of WMSD, consistent with existing literature that highlights the role physical activity has in enhancing musculoskeletal resilience and reducing occupational injury (23). Conversely, smoking was associated with an increased risk of WMSD. This may be attributed to smoking-induced vascular impairment, systemic inflammation, reduced bone mineral density, and heightened pain sensitivity (24–26). Additionally, smoking-related habits and stress may contribute to poor posture and increased muscle tension, which can further exacerbate the musculoskeletal strain.

Regarding occupational exposure factors, biomedical stressors, particularly the need to maintain static postures for extended periods of time, were also found to significantly increase the risk of WMSD. This finding concurs with prior research identifying prolonged static loading and repetitive movements in coal mining operations as principal risk factors for WMSD (27, 28). Muscle fatigue resulting from sustained postural demands can lead to chronic tension and microtrauma. Environmental exposures, such as working in cold or fluctuating temperatures, were similarly linked to increased WMSD prevalence. Miners operating in low-temperature conditions reported higher rates of musculoskeletal complaints, a trend supported by research indicating that cold environments amplify physical strain and reduce tissue elasticity, thereby increasing injury susceptibility (29). These findings highlight the importance of improving occupational and ergonomic assessments to prevent WMSD, particularly among smokers and those with long tenures.

Perceived health status is regarded as a comprehensive indicator reflecting an individual’s overall physiological, psychological, and functional state (30). This study found that perceived health status is one of the key indicators for predicting the risk of work-related musculoskeletal injuries: workers who self-reported poorer health had a significantly higher prevalence of musculoskeletal injuries compared to those who self-reported better health. This conclusion aligns with previous research findings among occupational populations. Previous studies indicate that among occupational groups such as healthcare workers and cleaners, individuals with poorer perceived health exhibit a significantly elevated risk of musculoskeletal symptoms, even after controlling for demographic characteristics and work-related factors (1, 30). From a mechanistic perspective, poorer perceived health may reflect diminished physical function, reduced musculoskeletal tolerance, or increased burden of underlying chronic conditions. These factors compromise an individual’s capacity to adapt to occupational exposures, such as prolonged physical labor, repetitive tasks, and poor work postures. Consequently, the decline in physiological reserves under sustained occupational stress may facilitate the onset of musculoskeletal injuries or exacerbate the persistence and worsening of existing WMSD symptoms.

From the perspective of occupational health practice and policy formulation, our findings hold significant implications. Among the multiple predictors identified in this study, certain factors—such as rest period scheduling, work posture, repetitive motions, and workplace environmental conditions—are highly amenable to intervention. This suggests that optimizing ergonomic design, rationally configuring work-rest systems, and improving workplace environments can effectively reduce the risk of WMSD occurrence at the practical level. Concurrently, the findings support integrating health promotion measures—such as encouraging physical activity and implementing tobacco control interventions—into occupational health management systems, with particular emphasis on targeted prevention for long-tenured employees and those in high-exposure positions. At the policy level, this study provides empirical evidence for developing and refining occupational hygiene standards, ergonomic guidelines, and labor protection policies, thereby advancing the implementation of prevention-oriented, comprehensive WMSD control strategies.

Results of our predictive modeling demonstrate that among the individual models built, NN and NB yields the most accurate predictions. The stacking ensemble model further improved overall performance, achieving an AUC of 0.759, which represents a 7.8% increase compared with the NN model. This substantial improvement can be attributed to the ability of the stacking approach to effectively integrate the prediction outputs of multiple base learners, thereby leveraging the complementary strengths of different modeling strategies in feature representation and decision-making. The stacking ensemble reduces the risk of overfitting associated with single models while substantially enhancing generalization performance. This conclusion concurs with recent studies in infectious disease, heart disease, breast cancer and lung adenocarcinoma, and peritoneal dialysis treatment predictions where stacking models demonstrated better performance (31–35).

Despite the notable performance advantages of stacking ensemble models, several practical considerations should be acknowledged when translating these approaches into real-world applications. Compared with single models, ensemble learning methods are generally associated with higher computational costs due to the need to train multiple base learners and a meta-learner. Moreover, their development and deployment require specific technological infrastructure and reliance on advanced technical skills related to machine learning model construction, parameter tuning, and performance evaluation. These factors may limit the widespread adoption of stacking models in routine occupational health practice. Future research should therefore focus on reducing computational complexity, optimizing ensemble strategies, and developing user-friendly tools to facilitate broader implementation across diverse application settings.

In addition to identifying key predictors, the machine learning models developed in this study have potential applicability in occupational health practice. Based on routinely available demographic, work-related, and health-related information, these models can be used to estimate individual-level risk of work-related musculoskeletal disorders (WMSDs), thereby facilitating early identification and risk stratification of high-risk workers. Recent study has demonstrated the feasibility of applying machine learning approaches for WMSD risk prediction and prevention-oriented occupational health management, supporting their use as decision-support tools rather than diagnostic instruments (36). Such models could be integrated into routine occupational health examinations or workplace health surveillance systems to support targeted preventive interventions, while further prospective validation is warranted to assess their generalizability and real-world performance.

To the best of our knowledge, our research is the first to use multiple machine learning algorithms to predict the risk of occurrence of WMSD in coal miners. Based on the six individual models constructed, an advanced stacking ensemble learning approach was further applied to effectively integrate the machine learning models, thereby enhancing overall predictive performance.

The study has a few limitations. Our study was conducted across two mining companies in one region of China. We have limited our study to this setting for pragmatic reasons in being able to access the participants and number needed. Furthermore, this epidemiologic study relied predominately on self-reported questionnaires, recall bias and other biases associated with questionnaire-based research cannot be avoided despite strict quality control during the survey process. The primary objective of this study was to identify key predictive factors based on a relatively large dataset, with the research focus placed on individual-level occupational exposure characteristics and health outcomes. Accordingly, the study site was not incorporated at the analytical level, which to some extent limited the assessment of potential heterogeneity between the two study locations. Future studies incorporating site-level information may further explore stratified or multilevel analytical approaches.

## 5 Conclusion

Our study confirmed that neck, shoulders, and lower back discomfort are highly prevalent among coal miners, underscoring the occupational burden of WMSD in this population. Numerous individual and work-related factors were significantly associated with the development of WMSD. These factors appeared to develop cumulatively over time. Importantly, our research demonstrated that machine learning algorithms can be used to predict WMSD risk with high accuracy. By integrating six base models using the stacking ensemble method, we achieved better predictive performance than any single model. This highlights the potential of ensemble learning to enhance the precision and reliability of risk prediction in complex, high-dimensional datasets.

## Data Availability

The raw data supporting the conclusions of this article will be made available by the authors, without undue reservation.
