# Supplementary material for: Determinants of work-related musculoskeletal disorders among coal miners in Jining, China: development of a predictive risk model
Source: Front Public Health. 2026 Feb 5;14:1729879. doi: 10.3389/fpubh.2026.1729879 (PMC12916718; doi:10.3389/fpubh.2026.1729879)
Supplement: Supplementary file 1 [file Table_1.docx]

**TableS1** Comparison of WMSDs among populations with different demographic characteristics

| Factors | N | Positive case(n) | Prevalence (%) | χ^2^ | *P* |  |
| --- | --- | --- | --- | --- | --- | --- |
| Gender |  |  |  |  |  |  |
| Male | 1308 | 1079 | 82.5 | 2.49 | 0.115 |  |
| Female | 101 | 77 | 76.2 |  |  |  |
| Age |  |  |  |  |  |  |
| ≤30 | 93 | 70 | 75.3 | 6.629 | 0.085 |  |
| 30~40 | 812 | 662 | 81.5 |  |  |  |
| 40~50 | 425 | 353 | 83.1 |  |  |  |
| >50 | 79 | 71 | 89.9 |  |  |  |
| BMI |  |  |  |  |  |  |
| ＜18.5 | 20 | 16 | 80 | 2.358 | 0.501 |  |
| 18.5~25 | 783 | 635 | 81.1 |  |  |  |
| 25~30 | 539 | 446 | 82.7 |  |  |  |
| ≥30 | 67 | 59 | 88.1 |  |  |  |
| Length of service in this job category | |  |  |  |  |  |
| <5 | | 415 | 325 | 78.3 | 10.793 | 0.013 |
| 5~10 | | 318 | 253 | 79.6 |  |  |
| 10~15 | | 351 | 299 | 85.2 |  |  |
| >15 | | 325 | 279 | 85.8 |  |  |
| Length of employment | |  |  |  |  |  |
| ≤10 | | 218 | 152 | 69.7 | 28.592 | <0.001 |
| 10~20 | | 877 | 731 | 83.4 |  |  |
| >20 | | 314 | 273 | 86.9 |  |  |
| Marital status | |  |  |  |  |  |
| Unmarried | | 66 | 43 | 62.5 | 13.471 | 0.001 |
| Married and living with spouse | | 1311 | 1087 | 82.9 |  |  |
| Married but separated | | 32 | 26 | 81.25 |  |  |
| Education |  |  |  |  |  |  |
| Junior high school and below | 104 | 83 | 79.8 | 1.962 | 0.743 |  |
| High school | 736 | 609 | 82.7 |  |  |  |
| junior college | 363 | 293 | 80.7 |  |  |  |
| bachelor degree | 202 | 167 | 82.7 |  |  |  |
| master degree or above | 4 | 4 | 100 |  |  |  |
| Physical exercise |  |  |  |  |  |  |
| No | 615 | 534 | 86.8 | 20.812 | <0.001 |  |
| Occasionally | 668 | 527 | 78.9 |  |  |  |
| 2～3times‎/month | 27 | 23 | 85.2 |  |  |  |
| 1～2times/week | 50 | 38 | 76 |  |  |  |
| > 2times/week | 49 | 34 | 69.4 |  |  |  |
| Smoking behavior |  |  |  |  |  |  |
| Non-smoker | 571 | 455 | 79.7 | 14.111 | 0.003 |  |
| Occasional | 361 | 285 | 78.9 |  |  |  |
| Regular | 433 | 380 | 87.8 |  |  |  |
| Quit smoking | 44 | 36 | 81.8 |  |  |  |

**TableS2** Comparison of WMSDs among different work-related factor groups

| Factors | N | Positive case | Prevalence (%) | χ^2^ | *P* |
| --- | --- | --- | --- | --- | --- |
| Perceived health status |  |  |  |  |  |
| Perfect | 462 | 308 | 66.7 | 111.438 | <0.001 |
| Fine | 832 | 741 | 89.2 |  |  |
| Poor | 103 | 96 | 93.2 |  |  |
| Very poor | 12 | 11 | 91.7 |  |  |
| Keep standing for long hours |  |  |  |  |  |
| Never | 236 | 181 | 76.7 | 38.375 | <0.001 |
| Occasionally | 253 | 185 | 73.1 |  |  |
| Frequently | 362 | 292 | 80.7 |  |  |
| Very Frequently | 558 | 498 | 89.2 |  |  |
| Keep kneeling for long hours |  |  |  |  |  |
| Never | 646 | 511 | 79.1 | 12.917 | 0.005 |
| Occasionally | 514 | 424 | 82.5 |  |  |
| Frequently | 175 | 152 | 86.9 |  |  |
| Very Frequently | 74 | 69 | 93.2 |  |  |
| Keep bending for long hours |  |  |  |  |  |
| No | 743 | 577 | 77.7 | 20.525 | <0.001 |
| Yes | 666 | 579 | 86.9 |  |  |
| Keep bowing the head for long hours |  |  |  |  |  |
| No | 786 | 603 | 76.7 | 34.235 | <0.001 |
| Yes | 623 | 553 | 88.8 |  |  |
| Keep bending knees for long hours |  |  |  |  |  |
| No | 751 | 582 | 77.5 | 22.573 | <0.001 |
| Yes | 658 | 574 | 87.2 |  |  |
| Repeat operation many times a working minute |  |  |  |  |  |
| No | 594 | 438 | 73.7 | 48.099 | <0.001 |
| Yes | 815 | 718 | 88.1 |  |  |
| Frequent repetition of the same movement on the back |  |  |  |  |  |
| No | 459 | 340 | 74.1 | 29.353 | <0.001 |
| Yes | 950 | 816 | 85.9 |  |  |
| Bending wrist up/down |  |  |  |  |  |
| No | 403 | 292 | 72.5 | 35.218 | <0.001 |
| Yes | 1006 | 864 | 85.9 |  |  |
| Work in uncomfortable postures |  |  |  |  |  |
| Never | 359 | 251 | 69.9 | 65.717 | <0.001 |
| Occasionally | 528 | 429 | 81.3 |  |  |
| Frequently | 333 | 303 | 91 |  |  |
| Very Frequently | 189 | 173 | 91.5 |  |  |
| Operate repetitively for multiple times per minute |  |  |  |  |  |
| Never | 281 | 203 | 72.2 | 56.769 | <0.001 |
| Occasionally | 429 | 327 | 76.2 |  |  |
| Frequently | 395 | 347 | 87.8 |  |  |
| Very Frequently | 304 | 279 | 91.8 |  |  |
| Carry heavy objects (more than 20 kg each time) |  |  |  |  |  |
| Never | 355 | 262 | 73.8 | 34.335 | <0.001 |
| Occasionally | 433 | 346 | 79.9 |  |  |
| Frequently | 331 | 289 | 87.3 |  |  |
| Very Frequently | 290 | 259 | 89.3 |  |  |
| Operate with hands or arms |  |  |  |  |  |
| Never | 224 | 166 | 74.1 | 46.305 | <0.001 |
| Occasionally | 289 | 210 | 72.7 |  |  |
| Frequently | 419 | 353 | 84.2 |  |  |
| Very Frequently | 477 | 427 | 89.5 |  |  |
| Overtime work |  |  |  |  |  |
| No | 725 | 549 | 75.7 | 40.49 | <0.001 |
| Yes | 684 | 607 | 88.7 |  |  |
| Feeling cold, wind or temperature change at work |  |  |  |  |  |
| No | 283 | 192 | 67.8 | 48.467 | <0.001 |
| Yes | 1126 | 964 | 85.6 |  |  |
| Enough rest time |  |  |  |  |  |
| No | 939 | 832 | 88.6 | 82.252 | <0.001 |
| Yes | 470 | 324 | 68.9 |  |  |
| Keep sitting for long hours |  |  |  |  |  |
| Never | 745 | 623 | 83.6 | 6.762 | 0.08 |
| Occasionally | 326 | 254 | 77.9 |  |  |
| Frequently | 186 | 149 | 80.1 |  |  |
| Very Frequently | 152 | 130 | 85.5 |  |  |
| Back position |  |  |  |  |  |
| Upright | 492 | 372 | 75.6 | 22.52 | <0.001 |
| slightly curved | 591 | 499 | 75.6 |  |  |
| Substantial bending | 326 | 285 | 87.4 |  |  |
| Keep turning for long hours |  |  |  |  |  |
| No | 779 | 618 | 79.3 | 8.695 | 0.003 |
| Yes | 630 | 538 | 85.4 |  |  |
| Neck position |  |  |  |  |  |
| Upright | 460 | 339 | 73.7 | 35.701 | <0.001 |
| slightly forward leaning | 601 | 507 | 84.4 |  |  |
| Large forward lean | 272 | 243 | 89.3 |  |  |
| head tilted back | 76 | 67 | 88.2 |  |  |
| Keep the neck in the same position for long hours |  |  |  |  |  |
| No | 613 | 475 | 77.5 | 15.29 | <0.001 |
| Yes | 796 | 681 | 85.6 |  |  |
| Keep turning the head for long hours |  |  |  |  |  |
| No | 889 | 702 | 79 | 15.5 | <0.001 |
| Yes | 520 | 454 | 87.3 |  |  |
| Keep wrist flexion for long hours |  |  |  |  |  |
| No | 623 | 471 | 75.6 | 31.461 | <0.001 |
| Yes | 786 | 685 | 87.2 |  |  |
| Hand position |  |  |  |  |  |
| No | 1141 | 947 | 83 | 3.701 | 0.054 |
| Yes | 268 | 209 | 78 |  |  |
| Turning around at the same time as bending over |  |  |  |  |  |
| No | 495 | 377 | 76.2 | 17.923 | <0.001 |
| Yes | 914 | 779 | 85.2 |  |  |
| Stretching or changing leg positions |  |  |  |  |  |
| No | 229 | 180 | 78.6 | 2.198 | 0.138 |
| Yes | 1180 | 976 | 82.7 |  |  |
| Lower extremities do the same movement frequently |  |  |  |  |  |
| No | 610 | 465 | 76.2 | 24.687 | <0.001 |
| Yes | 799 | 691 | 86.5 |  |  |
| Turn around often |  |  |  |  |  |
| No | 358 | 270 | 75.4 | 14.299 | <0.001 |
| Yes | 1051 | 886 | 84.3 |  |  |
| Carry heavy objects (more than 5kg each time) |  |  |  |  |  |
| Never | 297 | 228 | 76.8 | 31.076 | <0.001 |
| Occasionally | 376 | 288 | 76.6 |  |  |
| Frequently | 356 | 297 | 83.4 |  |  |
| Very Frequently | 380 | 343 | 90.3 |  |  |
| Holding onto something in hand. |  |  |  |  |  |
| No | 266 | 189 | 71.1 | 26.89 | <0.001 |
| Yes | 1143 | 967 | 84.6 |  |  |
| Performing the same type of work every day |  |  |  |  |  |
| No | 101 | 72 | 71.3 | 8.546 | 0.003 |
| Yes | 1308 | 1084 | 82.9 |  |  |
| Work requires socialization |  |  |  |  |  |
| No | 1143 | 936 | 81.9 | 0.098 | 0.755 |
| Yes | 266 | 220 | 82.7 |  |  |
| Complete work alternatively with co-workers |  |  |  |  |  |
| No | 418 | 351 | 84 | 1.498 | 0.221 |
| Yes | 991 | 805 | 81.2 |  |  |
| Working in the same workshop |  |  |  |  |  |
| No | 510 | 420 | 82.4 | 0.052 | 0.82 |
| Yes | 899 | 736 | 81.9 |  |  |
| Work changes everyday |  |  |  |  |  |
| No | 939 939 | 763 | 81.3 | 1.185 | 0.276 |
| Yes | 470 | 393 | 83.6 |  |  |
| Opening hours |  |  |  |  |  |
| <8h | 626 | 496 | 79.2 | 6.041 | 0.014 |
| >8h | 783 | 660 | 84.3 |  |  |
| Number of days on duty |  |  |  |  |  |
| <5days | 351 | 269 | 76.6 | 9.273 | 0.002 |
| >5days | 1058 | 887 | 83.8 |  |  |
| Shifting work |  |  |  |  |  |
| No | 337 | 289 | 85.8 | 8.821 | 0.066 |
| Two shifts | 14 | 13 | 92.9 |  |  |
| Three shifts | 859 | 685 | 79.7 |  |  |
| Four shifts | 56 | 49 | 87.5 |  |  |
| Others | 143 | 120 | 83.9 |  |  |
| Shortage of staff in the sector |  |  |  |  |  |
| No | 429 | 302 | 70.4 | 56.803 | <0.001 |
| Yes | 980 | 854 | 87.1 |  |  |
| Work for someone else |  |  |  |  |  |
| No | 972 | 774 | 79.6 | 12.401 | <0.001 |
| Yes | 437 | 382 | 87.4 |  |  |
| Use vibrator at work |  |  |  |  |  |
| Never | 456 | 350 | 76.8 | 26.438 | <0.001 |
| Occasionally | 425 | 338 | 79.5 |  |  |
| Frequently | 279 | 250 | 89.6 |  |  |
| Very Frequently | 249 | 218 | 87.6 |  |  |
| Driving the vehicle |  |  |  |  |  |
| Never | 566 | 450 | 79.5 | 16.291 | 0.001 |
| Occasionally | 397 | 320 | 80.6 |  |  |
| Frequently | 279 | 231 | 82.8 |  |  |
| Very Frequently | 167 | 155 | 92.8 |  |  |
| Work outdoors |  |  |  |  |  |
| No | 883 | 706 | 80 | 7.009 | 0.008 |
| Yes | 526 | 450 | 85.6 |  |  |
| Wrist on the edge of hard, angular objects |  |  |  |  |  |
| No | 728 | 567 | 77.9 | 17.689 | <0.001 |
| Yes | 681 | 589 | 86.5 |  |  |
| Take a break and start working again |  |  |  |  |  |
| No | 121 | 86 | 71.1 | 10.812 | 0.001 |
| Yes | 1288 | 1070 | 71.1 |  |  |
| Decide on your own work and rest |  |  |  |  |  |
| No | 1118 | 924 | 82.6 | 1.339 | 0.247 |
| Yes | 291 | 232 | 79.7 |  |  |
| Decide for yourself when to rest |  |  |  |  |  |
| No | 1050 | 875 | 83.3 | 4.65 | 0.031 |
| Yes | 359 | 281 | 78.3 |  |  |
| Rest time |  |  |  |  |  |
| ≤60min | 1183 | 987 | 83.4 | 10.066 | 0.002 |
| >60min | 224 | 167 | 74.6 |  |  |
| Rest breaks |  |  |  |  |  |
| ≤5times | 1296 | 1063 | 82 | 0.006 | 0.941 |
| >5times | 113 | 93 | 82.3 |  |  |

**TableS3** Results of multivariate logistic regression describing the relationship between personal and work-related factors with WMSD (Include age and gender)

| Factors |  | ORs | 95%CI | | *P* |
| --- | --- | --- | --- | --- | --- |
|  |  |  | Lower | Upper |  |
| Smoking behavior | No |  |  |  |  |
|  | Regular | 1.786 | 1.141 | 2.794 | 0.011 |
| Perceived health status | Perfect |  |  |  |  |
|  | Fine | 2.712 | 1.898 | 3.875 | <0.001 |
|  | Poor | 3.552 | 1.46 | 8.641 | 0.005 |
| Work in uncomfortable postures | Never |  |  |  |  |
|  | Occasionally | 1.806 | 1.112 | 2.933 | 0.017 |
| Repeat operation many times a working minute | No |  |  |  |  |
|  | Yes | 1.511 | 1.177 | 2.037 | 0.01 |
| Enough rest time | No |  |  |  |  |
|  | Yes | 0.583 | 0.391 | 0.87 | 0.008 |
| Keep bowing the head for long hours | No |  |  |  |  |
|  | Yes | 1.773 | 1.115 | 2.821 | 0.016 |
| Keep bending knees for long hours | No |  |  |  |  |
|  | Yes | 1.805 | 1.242 | 2.621 | 0.002 |
| Length of employment (years) | <10 |  |  |  |  |
|  | >20 | 2.092 | 1.056 | 4.143 | 0.034 |

**Table S4** Binary confusion matrix

| Actual | Predicted | |
| --- | --- | --- |
|  | Positive | Negative |
| Positive | True Positive (TP) | False Negative (FN) |
| Negative | False Positive (FP) | True Negative (TN) |

$$\begin{aligned} Accuracy=\frac{TP+TN}{TP+TN+FP+FN}\#\left( 1 \right) \end{aligned}$$

$$\begin{aligned} Precision=\frac{TP}{TP+FP}\#\left( 2 \right) \end{aligned}$$

$$\begin{aligned} Recall=\frac{TP}{TP+FN}\#\left( 3 \right) \end{aligned}$$

$$\begin{aligned} F1-score=2\times\frac{Precision\times Recall}{Precision+Recall}\#\left( 4 \right) \end{aligned}$$
